# Pumpkin (*Cucurbita moschata*) Fruit Extract Improves Physical Fatigue and Exercise Performance in Mice

**DOI:** 10.3390/molecules171011864

**Published:** 2012-10-09

**Authors:** Shih-Yi Wang, Wen-Ching Huang, Chieh-Chung Liu, Ming-Fu Wang, Chin-Shan Ho, Wen-Pei Huang, Chia-Chung Hou, Hsiao-Li Chuang, Chi-Chang Huang

**Affiliations:** 1 Department of Food and Nutrition, Providence University, Taichung 43301, Taiwan; 2 Graduate Institute of Athletics and Coaching Science, National Taiwan Sport University, Taoyuan 33301, Taiwan; 3 Department of Physical Education, Yuanpei University, Hsinchu 30015, Taiwan; 4 Graduate Institute of Sports Science, National Taiwan Sport University, Taoyuan 33301, Taiwan; 5 National Laboratory Animal Center, National Applied Research Laboratories, Taipei 11529, Taiwan; 6 School of Nutrition and Health Sciences, Taipei Medical University, Taipei 11031, Taiwan

**Keywords:** pumpkin, *Cucurbita moschata*, anti-fatigue, exercise performance, glycogen

## Abstract

Pumpkin (*Cucurbita moschata*) is a popular and nutritious vegetable consumed worldwide. The overall purpose of this study was to evaluate the effects of *C. moschata* fruit extract (CME) on anti-fatigue and ergogenic functions following physiological challenges. Male ICR mice from four groups designated vehicle, CME-50, CME-100 and CME-250, respectively (n = 8 per group in each test) were orally administered CME for 14 days at 0, 50, 100 and 250 mg/kg/day. The anti-fatigue activity and exercise performance were evaluated using exhaustive swimming time, forelimb grip strength, as well as levels of plasma lactate, ammonia, glucose, and creatine kinase after an acute swimming exercise. The resting muscular and hepatic glycogen was also analyzed after 14-day supplementation with CME. Trend analysis revealed that CME treatments increased grip strength. CME dose-dependently increased 5% body weight loaded swimming time, blood glucose, and muscular and hepatic glycogen levels. CME dose-dependently decreased plasma lactate and ammonia levels and creatine kinase activity after a 15-min swimming test. The mechanism was relevant to the increase in energy storage (as glycogen) and release (as blood glucose), and the decrease of plasma levels of lactate, ammonia, and creatine kinase. Therefore, CME may be potential for the pharmacological effect of anti-fatigue.

## 1. Introduction

“Agro Products” and food factors have been investigated as an important resource for postponing fatigue, accelerating the elimination of fatigue-related biomarkers and improving energy supply. For example, fermented rice bran [1], garlic [2], branched chain amino acids [3], or medium-chain fatty acids [4], and so on. Pumpkin (*Cucurbita moschata*) is a gourd-like squash of the genus *Cucurbita* and the family Cucurbitaceae. It is an economically important species cultivated worldwide, has high production, and is widely distributed in Taiwan. Pumpkin has received considerable attention in recent years because of the nutritional and health benefits of the bioactive compounds obtainable from its seeds and fruits. The chemical and pharmacological properties of *C . moschata* extracts from its stems, seeds and fruits have been investigated. These studies demonstrated that *C . moschata* has extensive bioactivities, such as hepatoprotection [5], anti-diabetes [6], anti-cancer [7], and anti-obesity properties [8]. Although more detailed and clinical studies are needed to confirm the medicinal effects in light of rational bioactivity function, *C . moschata* may be an anti-fatigue dietary supplement candidate. To this end we evaluated the anti-fatigue activities of ethanol extracts of *C . moschata* fruits in mice. We further examined the possible mechanism and provide evidence to support the ergogenic potential of *C . moschata* fruit extracts in the promotion of physical performance.

## 2. Results and Discussion

### 2.1. Body Weight, Skeletal Muscle Mass and Liver Weight

Morphological data from each experimental group are summarized in Table 1. There were no significant changes in the body, skeletal muscle mass (gastrocnemius and soleus muscles) and liver weights among vehicle, CME-50, CME-100 and CME-250 groups, and thus, the shot-term supplementation with CME treatments would not affect the body growth or enhancement of the weight of skeletal muscle.

**Table 1:** General characteristics of the experimental groups.

Characteristics	Vehicle	CME-50	CME-100	CME-250
Initial BW (g)	37.6 ± 0.6	37.7 ± 0.6	36.9 ± 0.4	36.9 ± 0.9
Final BW (g)	38.5 ± 0.3	40.4 ± 0.6	39.1 ± 0.6	39.0 ± 1.0
Skeletal muscle (g)	0.35 ± 0.01	0.37 ± 0.01	0.36 ± 0.01	0.36 ± 0.01
Liver (g)	2.00 ± 0.05	2.17 ± 0.08	2.00 ± 0.03	2.07 ± 0.09

Values are means ± SEM for n = 8 mice per group; Skeletal muscle mass contains both gastrocnemius and soleus muscles in the back part of the lower legs.

### 2.2. Effect of C. moschata Fruit Extract on the Clinical Biochemistry Tests

**Figure 1:**
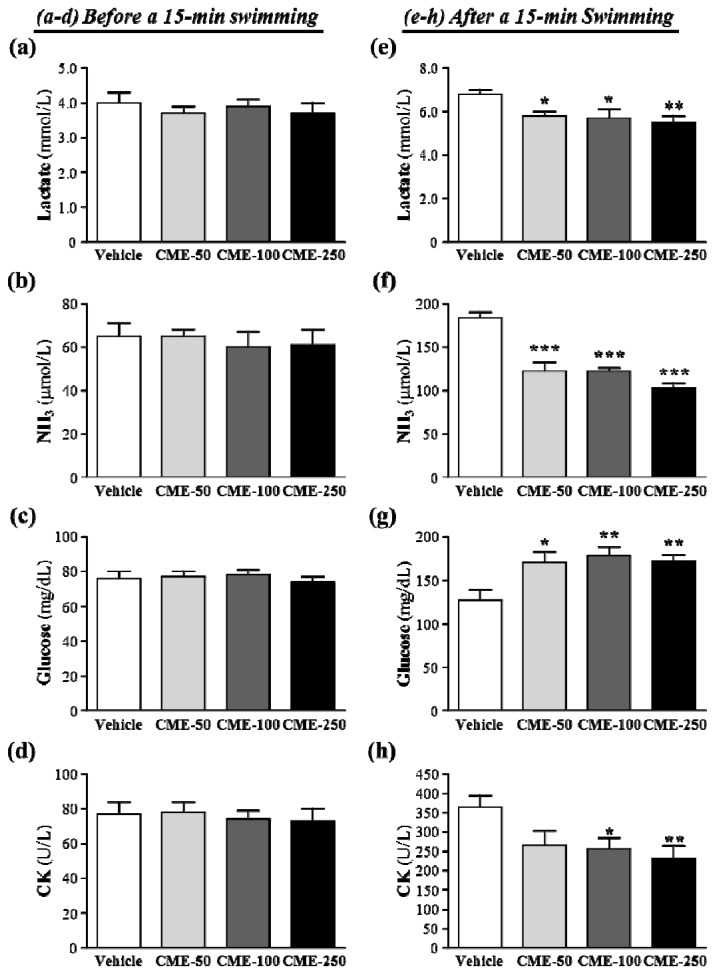
Effect of CME supplementation on plasma (**a**,**e**) lactate, (**b**,**f**) ammonia, (**c**,**g**) glucose levels and (**d**,**h**) creatine kinase (CK) levels before and after an acute exercise challenge. Mice were pretreated with vehicle, 50, 100, and 250 mg/kg of CME for 14 days, then 1 h later performed a 15-min swimming test without weight-loading. Data represent mean ± SEM of 8 mice in each group. * *p* < 0.05; ** *p* < 0.01; *** *p* < 0.001 compared to vehicle control.

The clinical biochemistry values of vehicle control and mice treated with CME-50, CME-100 and CME-250 were measured at the end of experiment, a 14-day oral feeding trial. As shown in the Table 2 and Figure 1d, there were no significant side effects in the liver profile (AST, ALT and albumin), bile duct function (ALP and total bilirubin), cardiac profile (LDH), muscular function (CK), renal profile (BUN, creatinine and uric acid), nutritional status (total protein), and lipid levels (TC and TG) among vehicle, CME-50, CME-100 and CME-250 groups. Moreover, the BUN level was significantly lower by 18.89% (*p* = 0.0367), 17.66% (*p* = 0.0497), and 22.57% (*p* = 0.0140) with CME-50, CME-100, and CME-250, respectively, than vehicle treatment. In the trend analysis, BUN level was decreased dose-dependently with the CME doses (*p* = 0.0049). Therefore, the acute toxicity study of CME at 50, 100 and 250 mg/kg administered orally to ICR mice did not caused any death or acute adverse effect on the clinical observation and mortality to the treatment mice. The results suggested that the supplementation with CME treatments should be safe for all test animals.

**Table 2:** Biochemical analysis of the CME treatment groups at the end of experiment.

Parameters	Vehicle	CME-50	CME-100	CME-250
AST (U/L)	83.9 ± 6.2	70.4 ± 4.4	83.0 ± 5.0	76.3 ± 8.5
ALT (U/L)	48.0 ± 2.8	45.6 ± 5.1	49.9 ± 2.8	43.9 ± 4.0
ALP (U/L)	120 ± 14	128 ± 16	137 ± 20	124 ± 13
LDH (U/L)	425 ± 34	429 ± 33	425 ± 31	362 ± 44
Albumin (g/dL)	2.7 ± 0.1	2.7 ± 0.1	2.8 ± 0.1	2.9 ± 0.1
TBIL(μg/dL)	55.2 ± 9.1	56.9 ± 5.6	59.6 ± 5.0	52.4 ± 3.2
TP (g/dL)	5.1 ± 0.1	5.1 ± 0.2	5.2 ± 0.2	5.2 ± 0.1
BUN (mg/dL)	25.5 ± 1.8 ^b^	20.7 ± 2.2 ^a^	21.0 ± 0.9 ^a^	19.7 ± 0.8 ^a^
Creatinine (mg/dL)	0.29 ± 0.01	0.26 ± 0.02	0.28 ± 0.01	0.28 ± 0.01
UA (mg/dL)	1.3 ± 0.1	1.1 ± 0.1	1.0 ± 0.1	1.1 ± 0.1
TC (mg/dL)	107 ± 6	110 ± 12	118 ± 5	107 ± 8
TG (mg/dL)	73.4 ± 10.5	71.3 ± 8.0	65.3 ± 5.9	60.0 ± 6.0

Values are means ± SEM for n = 8 mice per group; AST, aspartate aminotransferase; ALT, alanine aminotransferase; ALP, alkaline phosphatase; LDH, lactate dehydrogenase; TBIL, total bilirubin; TP, total protein; BUN, blood urea nitrogen; UA, uric acid; TC, total cholesterol; TG, triacylglycerol; Values in the same line with different superscripts letters (a, b) differ significantly, *p* < 0.05 by one-way ANOVA.

### 2.3. Effect of C. moschata Fruit Extract on Forelimb Grip Strength

**Figure 2:**
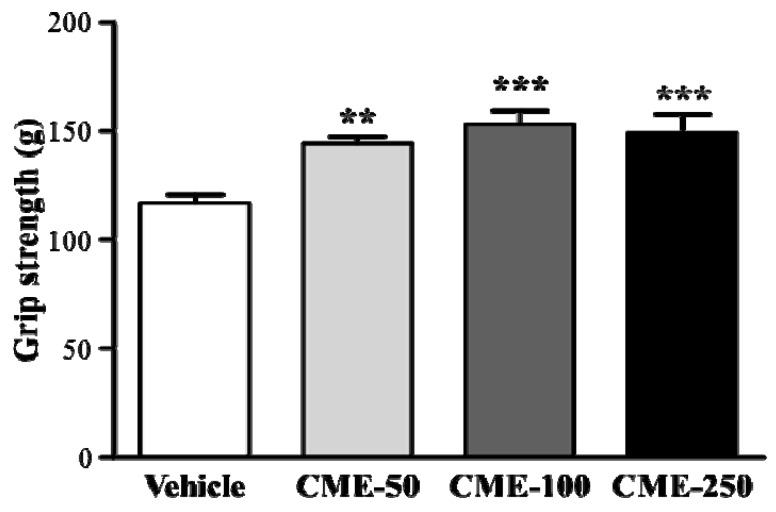
Effect of *C . moschata* extract (CME) supplementation on forelimb grip strength. Male ICR mice were pre-treated with vehicle, 50, 100, and 250 mg/kg ethanol extract of *C . moschata* (CME-50, CME-100, and CME-250) for 14 days, and underwent a grip strength test after 1 h of last administration. Data are presented as mean ± SEM of 8 mice in each group. ** *p* < 0.01; *** *p* < 0.001 compared to vehicle control.

As shown in the Figure 2, the data of grip strength was 116.9 ± 4.0, 144.1 ± 3.4, 153.1 ± 6.2, and 149 ± 8.8 g in the vehicle, CME-50, CME-100, and CME-250 groups, respectively, which was significantly higher, by 1.23 to 1.30-fold (*p* < 0.01), with CME treatments compared to vehicle control. In the trend analysis, grip strength was increased dose-dependently with the CME doses (*p* = 0.0006). Regulatory training program is needed for grip strength elevation, and the result showed that CME treatment has benefit for supporting grip strength.

### 2.4. Effect of C. moschata Fruit Extract on Exercise Performance in a Weight-Loaded Swimming Test

**Figure 3:**
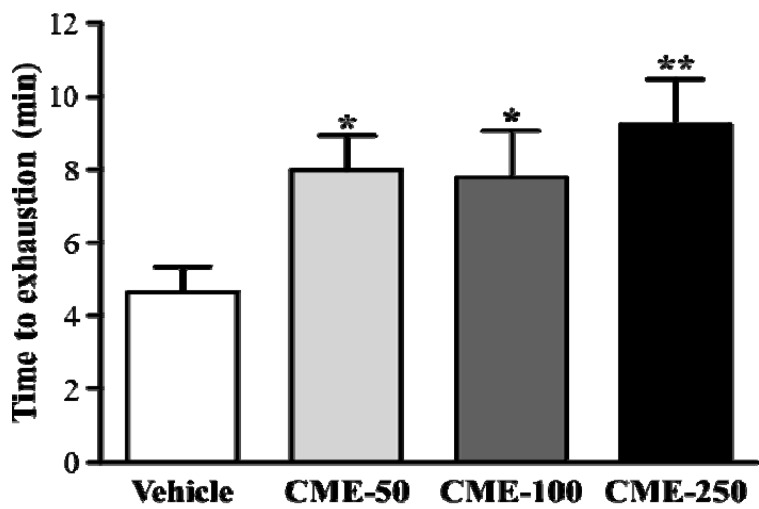
Effect of CME supplementation on swimming exercise performance. Mice were pretreated with vehicle, 50, 100, and 250 mg/kg of CME for 14 days and, then 1 h later performed an exhaustive swimming exercise with a 5% body-weight load attached to the mouse tail. Data represent mean ± SEM (n = 8 mice). * *p* < 0.05; ** *p* < 0.01 compared to vehicle control.

The energy metabolism of muscular activity determines the level of physiological fatigue [9]. Exercise endurance is an important variable in evaluating anti-fatigue treatment. In our study, the exercise endurance with a swim test in mice administered with vehicle, CME-50, CME-100, and CME-250 were 4.63 ± 0.69, 7.98 ± 0.94, 7.78 ± 1.28 and 9.24 ± 1.24 min, respectively, as shown in Figure 3. The swimming time was significantly longer by 1.72-fold (*p* = 0.0299), 1.68-fold (*p* = 0.0442), and 1.99-fold (*p* = 0.0034) with CME-50, CME-100, and CME-250, respectively, than vehicle treatment. In the trend analysis, maximal swimming time was increased dose-dependently with the CME doses (*p* = 0.0006).

### 2.5. Effect of Supplement with C. moschata Fruit Extract on Plasma Lactate, Ammonia, Glucose and CK Levels after an Acute Exercise Challenge

Biochemical variables, including lactate, ammonia, glucose, and CK, are important indicators of muscle fatigue after exercise [10]. Before exercise, we did not find statistical significant differences for these four biomarkers among four groups (Figure 1a–d). After an acute swimming exercise challenge, the levels of lactate, ammonia, glucose, and CK in the vehicle group were significantly increased by 1.72-fold (*p* < 0.0001), 2.82-fold (*p* < 0.0001), 1.67-fold (*p* = 0.0025), and 4.75-fold (*p* < 0.01), respectively, when compared with basal status as illustrated in the Figure 1e–h.

The muscle produces a great quantity of lactate when it obtains enough energy from anaerobic glycolysis during high-intense exercise. The increased lactate level further reduces pH value, which could induce various biochemical and physiological side effects, including glycolysis and phosphofructokinase and calcium ion release, through muscular contraction [11]. Lactate levels in the vehicle, CME-50, CME-100, and CME-250 groups were 6.8 ± 0.2, 5.8 ± 0.2, 5.7 ± 0.4 and 5.5 ± 0.3 mmol/L (Figure 1e) which were significantly lower with CME treatments, by 14.7% (*p* = 0.0318), 16.1% (*p* = 0.0267), and 19% (*p* = 0.0089) respectively, than vehicle treatment.

Ammonia, the metabolite of protein and amino acid, was linked to fatigue as early as 1922 [12]. An increase in ammonia in response to exercise can be managed by the use of glutamine and/or carbohydrates that interfere with ammonia metabolism [13]. The increase in ammonia level is related to both peripheral and central fatigue during exercise. Plasma ammonia levels in the vehicle, CME-50, CME-100, and CME-250 groups were 184 ± 6, 122 ± 10, 122 ± 4 and 103 ± 5 μmol/L, respectively. It was significantly lower, by 33.6–44%, with CME treatments, respectively, than vehicle treatment (*p* < 0.0001) (Figure 1f).

The energy supply for exercise initially came from the breakdown of glycogen, and energy will from circulating glucose released by the liver after intense exercise [14]. Therefore, blood glucose level is an important index for performance maintenance during exercise. The level of plasma glucose in the vehicle, CME-50, CME-100, and CME-250 groups were 127 ± 12, 170 ± 12, 178 ± 10, and 172 ± 2 mg/dL, respectively, and was significantly higher, by 1.34-fold (*p* < 0.05), 1.40-fold (*p* < 0.01), and 1.35-fold (*p* < 0.01), with CME-50, -100 and -250, respectively, compared to vehicle control (Figure 1g).

Plasma level of creatine kinase (CK) is a clinical biomarker for muscle damage, muscular dystrophy, severe muscle breakdown, myocardial infarction, autoimmune myositides and acute renal failure [15]. The CK activity in the vehicle, CME-50, CME-100, and CME-250 groups were 364 ± 30, 266 ± 37, 256 ± 28 and 232 ± 33 U/L, respectively (Figure 1h) and was significantly lower, by 30% (*p* = 0.0442) and 36.2% (*p* = 0.0157), with CME-100 and -250, respectively, than vehicle control. Therefore, CME supplementation should ameliorate skeletal muscle injury induced by acute exercise challenge.

By the trend analysis revealed whether the CME treatment had a significant dose-dependently effect. The statistic results shown the increase of dosage-dependence on blood glucose content (*p* = 0.0198) and decrease of dosage-dependence on plasma lactate and ammonia levels and CK activity with *p* = 0.0058, *p* < 0.0001, and *p* = 0.0113 respectively.

### 2.6. Effect of C. moschata Fruit Extract on Muscular and Hepatic Glycogen Levels

**Figure 4:**
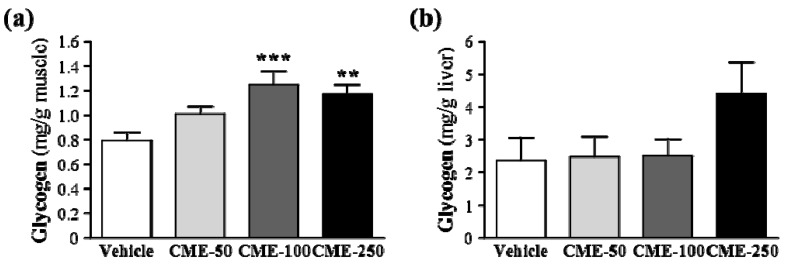
Effect of CME on (**a**) muscular and (**b**) hepatic glycogen levels at rest. Mice were pretreated with vehicle, 50, 100, and 250 mg/kg of CME for 14 days, all mice were sacrificed and examined for glycogen levels of muscle and liver tissues at 1 h after last treatment. Data represent mean ± SEM of 8 mice in each group. * *p* < 0.05; ** *p* < 0.01, *** *p* < 0.001 compared to vehicle control.

Energy storage and supply is another important factor related to exercise performance. In terms of energy expenditure with exercise, rapid ATP consumption and energy deficiency is a critical cause of physical fatigue [16]. Skeletal muscle mainly catabolizes fat and carbohydrates as sources of energy during exercise. Glycogen is the predominant source of glycolysis for ATP production. Therefore, glycogen storage directly affects exercise ability [17]. In Figure 4a, muscular glycogen levels in the vehicle, CME-50, CME-100, and CME-250 groups were 0.79 ± 0.07, 1.01 ± 0.06, 1.25 ± 0.11 and 1.17 ± 0.08 mg/g muscle, respectively. We found the significant increase in the glycogen contents of muscle tissues in CME-100 (*p* = 0.0003) and CME-250 (*p* = 0.0016) treatments. For the trend analysis, CME was found dose-dependently increased in the muscle tissue (*p* < 0.0001). In Figure 4b, hepatic glycogen levels in the vehicle, CME-50, CME-100, and CME-250 groups were 2.37 ± 0.69, 2.47 ± 0.62, 2.50 ± 0.51 and 4.40 ± 0.96 mg/g liver, respectively. Although we did not find any significant difference in the hepatic glycogen contents among groups, there was a slight increase in hepatic glycogen level in the CME-250 group compared to vehicle group (*p* = 0.0690). In the trend analysis, CME was found to increase hepatic glycogen levels in a dose-dependent manner (*p* = 0.0160).

Glucose and glycogen (the storage form of glucose) are the main sources of energy for exercise. The improvement of glucose storage in the muscles and liver is particularly important for endurance exercise. Glycogen is synthesized from glucose during glycogenesis and converted to glucose via glycogenolysis which is the reconversion process and provides a rapid extramuscular glucose supply. Also many hormones involved in regulation of blood glucose levels. For example, the insulin enables peripheral tissues to glucose uptake and glucagon stimulates liver glycogenolysis and gluconeogenesis to raise blood glucose concentration. In this study, we suppose that the improvement in carbohydrate metabolism seen in the mice treated with CME appear to be linked to the stimulation of glycogenesis at rest to act in a way similar to insulin. Therefore, it may help to explain that the elevated blood glucose levels in the CME-treated groups after a 15-min exercise challenge were all significantly higher when compared to the vehicle control group (Figure 1g).

On the other hand, ATP content in the muscle is another most important factor for deciding exercise performance. Thus, the regulation of CME treatment on muscular ATP levels in the further study is warranted.

### 2.7. Effect of C. moschata Fruit Extract on Hepatic and Muscular Tissues

**Figure 5:**
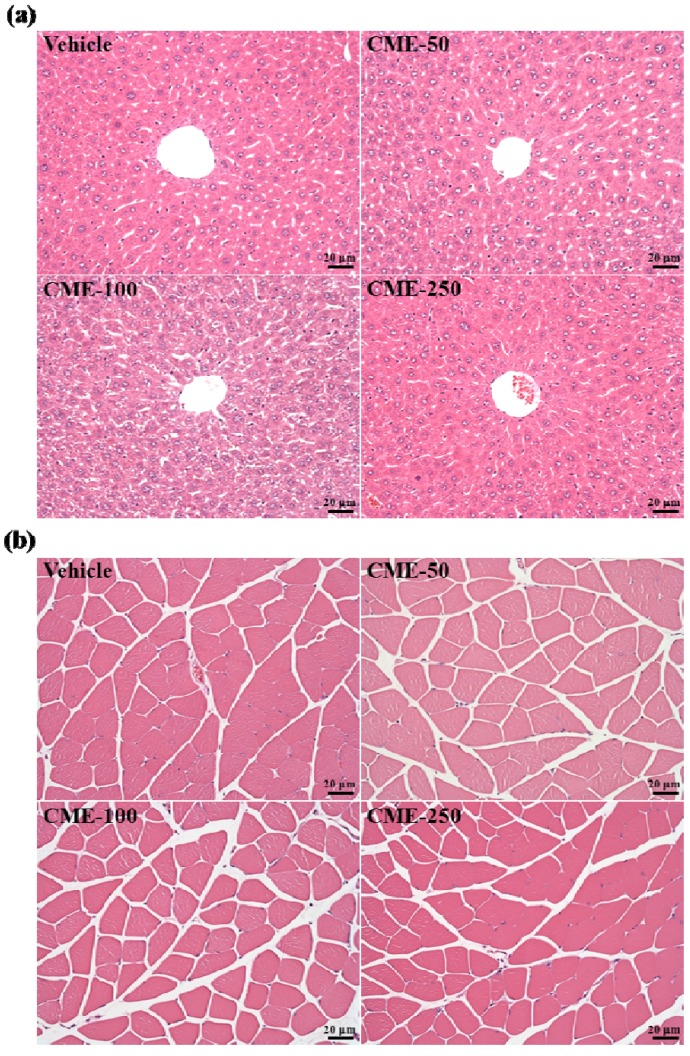
Effect of CME treatment on the morphology of liver (**a**) and skeletal muscle (**b**). Mice were pretreated with vehicle, 50, 100, and 250 mg/kg of CME for 14 days, all mice were sacrificed and examined for the morphology of liver and skeletal muscle at the end of experiment. Specimens were photographed with a light microscope (Olympus BX51; Olympus Co., Ltd., Tokyo, Japan). (H&E stain, magnification: ×200, Scale bar, 20 μm).

We also examined whether CME treatments could cause any negative effect on liver or skeletal muscle tissues of healthy mice. We examined plasma aminotransferase (AST and ALT) and CK activities (Table 2 and Figure 1d), hepatic and muscular morphology in CME-treated mice (Figure 5) and found no indication of a deleterious effect from CME treatments.

## 3. Experimental

### 3.1. Plant Material and Extraction

Fresh pumpkin (*C. moschata*, CM), fruits were purchased from a local market. Before extraction from the fruits, the seeds were removed and raw fruits with skin were cut with a slicer, and dried by solar drying. The dehydrated fruits were ground into powder. The dried powder (230 g) was extracted three times with 95% ethanol (2 L, 25 °C, 1 week). After filtration, the solvent was concentrated by use of a rotary evaporator (Büchi R-215) to obtain the ethanol extract (26.1 g). The ethanolic extract of *C. moschata* (CME) was used for animal experiments in this study. A voucher specimen of *C. moschata* was deposited at the Graduate Institute of Sports Science, National Taiwan Sport University (Taoyuan, Taiwan).

### 3.2. Animal and Experiment Design

The specific pathogen-free (SPF) male Institute of Cancer Research (ICR) strain mice (12-week-old) were purchased and provided from BioLASCO (A Charles River Licensee Corp., Yi-Lan, Taiwan). Before experiments, the mice were raised for one week to adapt the environment and diet. All animals were given a standard laboratory diet (no. 5001; PMI Nutrition International, Brentwood, MO, USA), distilled water *ad libitum* and housed at room temperature (24 ± 1 °C) with a 12-h light/12-h dark cycle (lights on from 6:00 am to 6:00 pm) and humidity 50–60%. All animal experiments conformed to the guidelines of the Institutional Animal Care and Use Committee (IACUC) of National Taiwan Sport University (NTSU). This study under the protocol IACUC-10110 was approved by the IACUC ethics committee. Mice were separated into four groups (n = 8 per group in each test) for treatment: (1) vehicle control; (2) 50 mg/kg CME (CME-50); (3) 100 mg/kg CME (CME-100); (4) 250 mg/kg CME (CME-250). Vehicle or CME was administrated by oral gavage. The control group received the vehicle, distilled water, at the same dosage volume of 10 mL/kg throughout the same period.

### 3.3. Forelimb Grip Strength

For measurement of forelimb grip strength in mice, a low-force testing system (Model-RX-5, Aikoh Engineering, Nagoya, Japan) was applied in this test. Each mice with different treatments was measured the amount of tensile force by using the force transducer equipped with a mental bar (2 mm in diameter and 7.5 cm in length). As described in our previous study [18], we grasped the mouse at the base of the tail and lowered it vertically toward the bar. The mouse was pulled slightly backwards by the tail while the two paws (forelimbs) grasped the bar, which triggered a “counter-pull”. The grasping force was recorded by grip strength meter in grams. The mice were trained to be familiar this procedure for three days before CME administration. These four groups didn’t show the significant difference before CME administration. After consecutive administration and one hour of last treatment, the test of grip strength was performed. The maximal force (in grams) induced by the counter-pull of mice forelimbs was used as grip strength.

### 3.4. Swimming Exercise Performance Test

The swimming exercise performance test was used as previously described with some modifications [18]. Mice were pre-treated with vehicle, CME-50, CME-100, and CME-250 mg/kg for continued 14 days and one hour after the last administration, followed by an exhaustive swimming test. The mouse was taken out from each treatment for swimming exercise and loaded the constant weight (lead fish sinkers, attached to the tail) corresponding to 5% of individual body weight. The mice were individually carried out in a columnar swimming pool (65 cm tall and radius 20 cm) with 40-cm water depth maintained at 27 ± 1 °C. The endurance for each mouse was measured as swimming times recorded from the beginning to exhaustion which was determined by observing loss of coordinated movements and failure to return to the surface within 7 s. In the swimming period, it was considered the time spent floating, struggling and making necessary movements until exhaustion and possible drowning.

### 3.5. Determination of Blood Biochemical Variables

The effects of CME on plasma lactate, ammonia and glucose levels, and creatine kinase (CK) activity were evaluated after exercise. After 1 h of the last administration, a 15-min swimming test was performed without weight-loading. Blood samples were immediately collected from the submandibular duct of pre-treated mice after swimming exercise. The plasma was prepared by centrifugation at 1,500 × *g*, 4 °C for 10 min. Lactate, ammonia, and glucose levels and CK activity were determining by use of an auto-analyzer (Hitachi 7060, Hitachi, Tokyo, Japan). Other biochemical analyses were determined with use of an automatic analyzer (Hitachi 7080).

### 3.6. Tissue Glycogen Determination

Since liver and skeletal muscles are the two major tissues for glycogen deposition, we investigated whether glycogen contents of these two target tissues could increase by CME administration. The mice were pretreated with vehicle, CME-50, CME-100, and CME-200 for continued 14 days. One hour after the last treatment, all mice were killed. The liver and muscle were excised and weighed for following glycogen level analysis. The method of glycogen analysis as previously describe was applied in this study with some modifications [19]. For each mouse, 100 mg of liver and muscle was finely cut, weighed and homogenized in 0.5 mL cold 10% perchloric acid. After centrifuge of 15 min with 15,000 × *g* at 4 °C, the supernatant was carefully decanted and kept on ice for analysis. A standard glycogen (Sigma) or tissue extract, 30 μL, was added to 96-well micro-plates, and iodine-potassium iodide reagent, 200 μL, was added to each well for iodine binding to glycogen. An amber-brown compound developed immediately after the reaction. ELISA reader (Tecan Infinite M200, Tecan Austria, Salzburg, Austria) with wavelength 460 nm measured the absorbance after material rested for 10 min.

### 3.7. Histological Examination of Liver and Skeletal Muscle Tissues

Liver and skeletal muscle tissues were removed from vehicle and CME treatment groups at the end of experiment, andfixed in 10% buffered formalin and then embedded in paraffin. Paraffin-embedded samples were sectioned (3 μm) and underwent hemotoxylin and eosin (H&E) staining as described in our previous report [20].

### 3.8. Statistical Analysis

Data are expressed as mean ± SEM, analyzed by one-way AVOVA and A Cochran-Armitage test for dose-effect trend analysis with SAS version 9.0 (SAS Inst., Cary, NC, USA). *p* < 0.05 was considered statistically significant.

## 4. Conclusions

The contents of nutrient in pumpkin contain water, fat, protein, carbohydrate, fiber, ash *etc*. [21]. It also provides not only the important minerals including calcium, phosphorous, iron, magnesium, potassium, sodium but also the rare elements including zinc, selenium, copper, cobalt, nickel, chromium, and nicotinic acid. The functional activities of famous phytocompounds of pumpkin including tocopherol (e.g., α- and γ-tocopherol), carotenoid (e.g., β-carotene, β-cryptoxanthin, lutein, and zeaxanthin), and β-sitosterol has been reported for anti-inflammation [22], anti-oxidation [23], anti-carcinogenic activity [24], and anti-angiogenesis [25]. In recent studies, the new secondary metabolites, dehydrodiconiferyl alcohol and tetrasaccharide glyceroglycolipid, of pumpkin showed anti-lipogenic effect [8] and glucose-lowing activity [6]. However, to our best knowledge, to date there is no report on the testing of in vivo bioefficacy of pumpkin to anti-fatigue agents.

In conclusion, our data suggest that the ethanolic extract of the fruits of *C. moschata* (CME) could increase the swimming time to exhaustion of test animals, as well as increase plasma glucose and the muscular and hepatic glycogen levels, and decrease the plasma lactate and ammonia levels. These results indicate that CME has anti-fatigue activity and can elevate exercise performance. Although the exact bioactive phytocompounds and detailed anti-fatigue mechanisms of *C. moschata* remain to be elucidated, this study provides science-based evidence to support that *C. moschata* could be a promising anti-fatigue agent and an ergogenic aid.
